# *Artemisia vulgaris* efficacies against various stages of *Aedes aegypti*

**DOI:** 10.14202/vetworld.2020.1423-1429

**Published:** 2020-07-24

**Authors:** Vika Ichsania Ninditya, Endah Purwati, Ajeng Tyas Utami, Aprillyani Sofa Marwaningtyaz, Nadia Khairunnisa Fairuz, Rini Widayanti, Penny Humaidah Hamid

**Affiliations:** 1 Faculty of Veterinary Medicine, Universitas Gadjah Mada, Yogyakarta, Indonesia; 2 Study Program, Faculty of Pharmacy, Universitas Gadjah Mada, Yogyakarta, Indonesia

**Keywords:** *Aedes aegypti*, *Artemisia vulgaris*, bioinsecticide

## Abstract

**Background and Aim:**

*Aedes aegypti* is the vector of dengue fever, dengue hemorrhagic fever, chikungunya, and, most recently, Zika. Dengue fever is one of Indonesia’s endemic diseases. The principal tool for preventing dengue is controlling *Ae. aegypti* by chemical insecticides since vaccine against dengue is still under research. However, *Ae. aegypti* developed resistance to various chemical insecticides worldwide. Therefore, research on alternate compounds as mosquito insecticides is urgently needed. This study demonstrated the efficacy of *Artemisia vulgaris* extract as larvicidal, ovicidal, adulticidal, repellency, and oviposition deterrent activity against *Ae. aegypti*.

**Materials and Methods:**

*A. vulgaris* was obtained from Temanggung, Indonesia, while the eggs of *Ae. aegypti* were collected from Yogyakarta, Indonesia, and were hatched in Laboratory of Parasitology, Faculty of Veterinary Medicine, Universitas Gadjah Mada. Larvicidal activity was evaluated according to the WHO protocol; adulticidal activity was performed using the Centers for Disease Control protocol. Oviposition activity was evaluated using ovitraps added with *A. vulgaris* extract, complete protection time in the repellent assay was defined as the number of minutes elapsed between compound application and the landing of the first mosquito.

**Results:**

A test of the larvicidal activity of *A. vulgaris* extract returned an LC_50_ of 65.8 ppm (r^2^=0.9014) in 1 h and 18.6 ppm (r^2^=0.575) in 24 h. *A. vulgaris* was effective as an adulticidal, demonstrating LC_50_ values of 11.35 mg (r^2^=0.875) in 90 min, 9.63 mg (r^2^=0.924) in 105 min, and 6.46 mg (r^2^=0.925) in 120 min. *A. vulgaris* at a concentration of 1000 ppm was able to reach 96% of oviposition deterrent effect. The ovicidal assay, a concentration of 1000 ppm resulted in 82.67% of eggs remaining unhatched. An extract concentration of 80 mg/ml achieved 63.3±3.5% biting repellency in adults.

**Conclusion:**

This study gives a clear indication that *A. vulgaris* extract acts on *Ae. aegypti* at various developmental stages and is a potential alternative bioinsecticide for controlling this disease vector.

## Introduction

Mosquito-borne diseases are endemic to more than 100 countries, resulting in the deaths of 2 million people every year and placing as many as 2100 ­million people around the world at risk [1]. More people in recorded history have died from diseases transmitted by mosquitoes than from fighting in all the wars combined [2]. Dengue fever is the most common ­mosquito-borne disease, affecting a wide spectrum of the global population [3]. It is common knowledge that mosquito-borne diseases are endemic to Indonesia, and the highest frequency of outbreaks occurring annually is those of dengue fever [4]. No specific treatment or vaccine for dengue has been found so far [5]. An extraordinary outbreak was reported to affect most of Indonesia, including 11 provinces, 12 districts, and three municipalities, with 8487 infection cases followed by 108 deaths in January 2016-February 2016. Patients aged 5-14 years were predominantly affected, comprising 43.44% of the total reported cases. The only available preventive measure against dengue virus transmission is the control of the disease vector [1]. The eradication of the disease vector is difficult in Indonesia, as in any other tropical country, because the climate supports the mosquito’s life cycle. Moreover, global warming contributes significantly to the expanding mosquito population carrying dengue fever, yellow fever, malaria, and many other diseases that pose a risk to humans [6]. Vector control programs employing chemical and synthetic insecticides have long been utilized to prevent the transmission of mosquito-borne diseases. The use of chemical insecticides over a long period results in a multitude of problems, such as insecticide resistance, environmental pollution, and adverse impacts on humans and other organisms [7]. Botanical insecticides are considered to be environmentally-friendly and safe for other organisms [8]. The use of botanical remedies in control programs has been limited thus far; hence, no studies have shown vector resistance to botanical-based insecticides [9]. Plant extracts or phytochemicals are potential sources of commercial anti-mosquito bioactive compounds. Some phytochemical substances act as general toxicants against the adult and larval stages, while others act as repellents or attractants that interfere with the growth and development through the production of olfactory stimuli [1].

The genus *Artemisia* is one of the largest groups in the *Asteraceae* family, consisting of more than 800 species with a widespread global distribution [10]. Many of the identified *Artemisia* species grow in Asia, Europe, North and Central America, and Northern Africa [11]. *Artemisia vulgaris* is known locally as mugwort. Its essential oils are used as insecticidal, antimicrobial, and antiparasitic agents; fumigants; repellents of *Musca domestica*; hepatoprotectants; and analgesic agents [11]. Another species of *Artemisia*, *Artemisia herba-alba* was also reported to act as a vermifuge by reducing the egg and worm load of *Heterakis gallinarum* eggs in infected birds [12] and *Haemonchus contortus* [13]. The use of the herb *A. absinthium*, also known as wormwood, was also reported in ancient Egyptian times as being active against vermin, a common word for vector pests [14], and evidently exhibited strong larvicidal activities toward mosquitoes [15]. *Artemisia* is a very well-known artemisinin compound that effectively reduces the malarial parasite burden. In addition, in the whole dried form, it acts synergistically to overcome malarial resistance to a single active compound [11,16].

This study aimed to analyze the ovicidal, larvicidal, adulticidal, oviposition, and repellent activities of an *A. vulgaris* extract originating from Indonesia against *Aedes aegypti* mosquitoes.

## Materials and Methods

### Ethical approval

All of the experimental procedures reported herein were approved by the Ethics Committee for pre-clinical research of LPPT Universitas Gadjah Mada, Yogyakarta, under approval number 00076/04/LPPT/VI/2017. Ethical clearance related to the human skin test (repellency assay) was issued by the committee of the Faculty of Medicine, Universitas Gadjah Mada, under approval number KE/FK/0622/EC/2017.

### Plant materials

*A. vulgaris* was collected in March 2017 from Temanggung, Central Java Province, Indonesia, which is located at 7°19′30′′ latitude and 110°14′88′′ longitude. The identification of species was issued by the Department of Plant Systematic, Universitas Gadjah Mada, Yogyakarta, Indonesia. A total of 5 kg *A. vulgaris* were cleaned and dried using an oven at 55°C for 7 days. Dried leaves were ground into a powder in a grinding machine. Then, ethanol 95% was added to the powder for *A. vulgaris* extraction. The mixture was homogenized for 30 min and incubated for 1 day at room temperature. Complete removal of the filtrate was accomplished in a vacuum rotary evaporator. The extract was heated in a water bath at 70°C. The final extract was obtained in extruded form and stored at 4°C until use in further experiments. One gram of the extract was dissolved in 100 ml of ethanol to prepare a 1% stock solution [17,18].

### Egg collection and mosquito rearing

Eggs of *Ae. aegypti* were collected from several areas in Yogyakarta, Indonesia, using 200 ovitraps. The eggs were transferred to the Laboratory of Parasitology, Faculty of Veterinary Medicine, Universitas Gadjah Mada, Yogyakarta, for hatching. The eggs were placed in 20×15×5 cm plastic containers filled with 500 ml tap water. Larvae were fed with chicken liver and maintained at 28°C and 70%-85% relative humidity, with a photoperiod of 12 h light and 12 h dark. The eggs hatched within ±24 h. The larvae developed into the third instar stage in ~4 days. Feeding was continued until the larvae transformed into pupae. Pupae were collected and transferred to glass beakers filled with 500 ml of water [19].

### Larvicidal assay

A larvicidal assay was performed using late third instar and early fourth instar larvae of *Ae. aegypti*. The larvicidal activity was evaluated according to the WHO protocol [20], as outlined below. Five larvae were transferred to a cup filled with 25 ml of distilled water as a negative control; various concentrations of *A. vulgaris* extract (1, 5, 10, 50, 100, 500, and 1000 ppm). Since temephos is known as common chemical larvicidal usually used [21], we used temephos as a positive control at concentrations similar to those of *A. vulgaris* extract. Three replicates were performed for each concentration, and larval mortalities were counted after 1 h and 24 h of exposure.

### Maintenance of adult mosquitoes

On emergence from pupae, mosquitoes were transferred to 20×20×20 cm mosquito cages and fed with 10% sucrose solution until the time of testing [22].

### Centers for disease control (CDC) bottle bioassay

The CDC bioassay was performed to determine the time required for the insecticide to penetrate a mosquito. The doses applied in this study were based on the CDC guidelines [23] as follows: 1.25 μg, 2.5 μg, 5 μg, and 10 μg for cypermethrin as positive controls; the ethanol bottle for the negative control; and *A. vulgaris* at concentrations of 10 μg, 50 μg, 100 μg, 500 μg, 1000 μg, 5000 μg, 10,000 μg, 50,000 μg, and 100,000 μg. Lower concentrations of *A. vulgaris* were not used. The CDC bottles were coated according to the CDC protocol [23]. The quantity of *A. vulgaris* extract was expressed as a mass because, following dilution in ethanol, the coated bottles were dried [23]. Between 10 and 20 unfed mosquitoes aged from 2 to 5 days were removed by aspiration and gently blown into the bottle. Susceptibility tests were performed according to the CDC protocol, in three replicates. Alive and dead mosquitoes were enumerated at the following intervals: 15, 30, 35, 40, 45, 60, 75, 90, 105, and 120 min. “Dead” mosquitoes are mosquitoes that cannot stand and slide along the curvature of the bottle [23]. Total mortality in percent (Y-axis) versus time (X-axis) was then analyzed for all replicates [24].

### Oviposition

Oviposition activity was evaluated using ovitraps filled with 100 ml of tap water. Then, *A. vulgaris* extract was added to these ovitraps to obtain final concentrations of 100 ppm, 500 ppm, and 1000 ppm. Ovitraps containing 1 ml ethanol served as controls. Ovitraps filled with test and control solutions were placed in a mosquito cage containing 50 blood-fed females. Three replicates were performed for each concentration. Eggs were collected daily until no eggs were laid for at least 48 h. The eggs were counted under a dissecting microscope [9]. Effective repellency (EF) was calculated using the following formula:


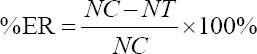


while the oviposition activity index (OAI) was calculated using the following formula:


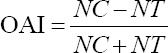


NC is the total number of eggs laid on the control paper. NT is the total number of eggs laid on the treatment paper [25]. Values of OAI +0.3 or above were considered to indicate an attractant and those −0.3 or below, a deterrent [26].

### Ovicidal assay

One blood-fed female was transferred to a cup and allowed to lay eggs. For *Ae. aegypti*, the bottom of the cup was lined with filter paper on wet cotton (provided as an oviposition site). After 2 days, the eggs were collected and counted under a dissecting microscope. The filter papers yielded minimum 100 eggs per piece cut for further process. The eggs were submerged in three different concentrations of *A. vulgaris* extract: 100 ppm, 500 ppm, and 1000 ppm. Ethanol served as a control. After 5 h of exposure, the eggs from each replicate were transferred to a different container filled with water for the hatching process. The hatched larvae were collected and counted daily until no larvae hatched for at least 48 h. Hatched larvae were counted, and three replicates were performed for each concentration [9].

### Repellent assay

One milliliter (mg/ml) of each test solution was smeared on the dorsal side of one hand (wrist to fingertips) of the subject. The concentration units of this part of the experiment were adjusted according to the WHOPES [27]. Thirty minutes after application, the hand was placed up to the wrist inside the repellent chamber through a hole for 3 min to allow the female mosquitoes to bite the subject. The tests were repeated at intervals of 30 or 60 min. The landing of one mosquito during the 3-min test interval concluded the test for each repellent dose [28]. Complete protection time was defined as the number of minutes elapsed between compound application and the landing of the first mosquito. All experiments were performed in triplicate [5,27].

### Statistical analysis

Data were analyzed with GraphPad software (GraphPad Inc., USA), including the determination of dose-response curves. The dose-response curve itself was defined by non-linear regression model of logarithmic dosage (X) against responses (Y). The lethal concentrations 50 (LC_50_) and LC_100_, concentration, which causes the death of 50% and 100% of the tested mosquitoes, were drawn from the curve by GraphPad software.

## Results

**Figure-1:**
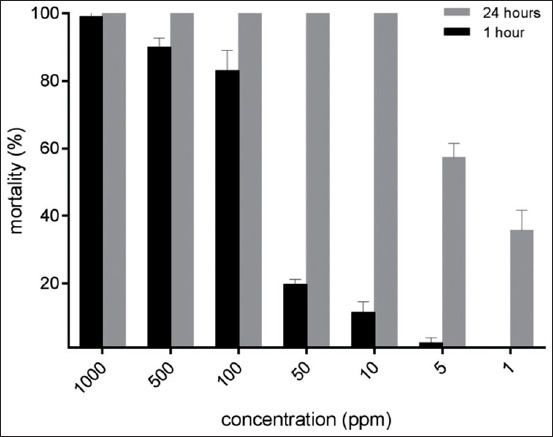
Larvicidal activity of *Artemisia vulgaris* against the tested *Aedes aegypti*.

The larvicidal activity of *A. vulgaris* extract at different concentrations, i.e., 1, 5, 10, 50, 100, 500, and 1000 ppm, is presented in Figure-1. At a concentration of 1000 ppm, the application of the extract resulted in 100% larval mortality in the 1^st^ h of observation. Total larval mortality was also achieved with the 10 ppm concentration after 24 h of incubation. The *A. vulgaris* extract affected larval mortality in a concentration-dependent manner. Based on the mortality rate, the calculated LC_50_ values of *A. vulgaris* extract were 65.8 ppm (r^2^=0.9014) in 1 h and 18.6 ppm (r^2^=0.575) in 24 h. In addition, increasing the incubation period from 1 to 24 h significantly, by multiple t-tests, enhanced the extract’s effect at 500 ppm (p≤0.0001), 100 ppm (p≤0.0001), 50 ppm (p≤0.0001), 10 ppm (p≤0.0001), 5 ppm (p≤0.01), and 1 ppm (p≤0.1).

**Figure-2:**
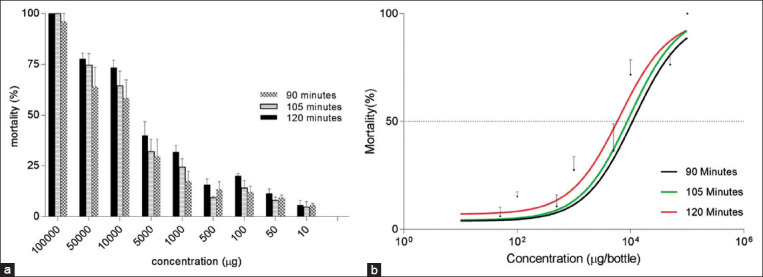
(a) Adulticidal activity of *Artemisia vulgaris* against the tested *Aedes aegypti*. (b) Dose-response analysis of the adulticidal effect of *Artemisia vulgaris*.

The activity of *A. vulgaris* against the adult stage of *Ae. aegypti* is presented in Figure-2a and b. The insecticidal effect of the extract against adult mosquitoes was also influenced by exposure time. A dose-response curve (Figure-2b) showed that the LC_50_ values of *A. vulgaris* extract against adult *Ae. aegypti* were 11.35 mg (r^2^=0.875) in 90 min, 9.63 mg (r^2^=0.924) in 105 min, and 6.46 mg (r^2^=0.925) in 120 min. However, no significant difference was found (p≤0.5) between the tested concentrations at any fixed exposure time (90, 105, or 120 min) in this experiment. No mortality was observed in the control group during the observation period.

**Figure-3:**
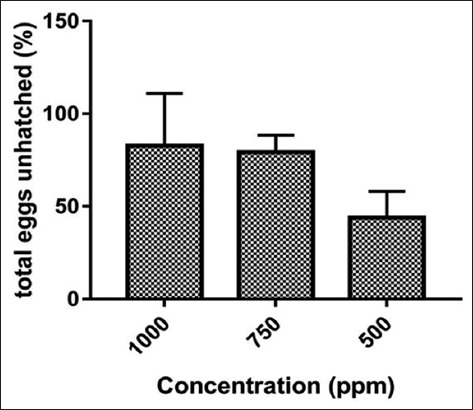
Ovicidal activity of *Artemisia vulgaris* to *Aedes aegypti*.

The ovicidal assay demonstrated that *A. vulgaris* in the concentration of 1000 ppm resulted in only 82.67% eggs unhatch (Figure-3). Ovicidal activity is 79.33% at a concentration of 750 ppm. In the concentration of 500 ppm, ovicidal activity is 44% (Table-1).

**Table-1:** Oviposition deterrent activity of *Artemisia vulgaris.*

Concentration (ppm)	Number of eggs		ER (%)	OAI

	Treated	Control		
1000	168	3900	96	−0.9174
500	731	3970	82	−0.689
100	1197	4780	75	−0.5995

In this experiment, *A. vulgaris* showed a stronger repellent activity than the positive control during the observation period (Table-2). The tested concentrations were 20, 40, and 80 mg/ml, which were lower than that of the positive control, i.e., a commercial cream containing N,N-Diethyl-meta-toluamide as 12%.

**Table-2:** Repellent activity of *Artemisia vulgaris* extract.

Concentration	Percent of repellency±SD					

	Treated			Positive control		
	
	30 min	60 min	90 min	30 min	60 min	90 min
20 mg/ml	58±6	27±23.5	1.7±2.9	41.3±32.3	46.7±30.5	56.3±18.5
40 mg/ml	65.3±13.3	35±11.1	21.3±27.1	26.3±25.1	49±17.0	62.3±16.6
80 mg/ml	63.3±3.5	39.7±32.7	26±23.9	2.7±4.6	10.3±17.9	15.7±27.1

## Discussion

Insecticide resistance in Southeast Asia follows an increasing trend [29-35], mirroring the status of other types of resistance worldwide [36-41]. Resistance development is a serious problem since arthropod-borne diseases are largely controlled by insecticide-based vector action. To minimize the use of chemical compounds, natural herbs may serve as potential insecticides in the control of disease vectors, considering their abundance and safety. In this experiment, we evaluated *A. vulgari*s extract for its efficacy against the mosquito larval and adult stages, oviposition deterrent/attractant activities, ovicidal effect, and repellent activity. *A. vulgaris* showed prominent effects on *Ae. aegypti* stages imply its the potential use as non-chemical-based insecticide.

The larvicidal LC_50_ of *A. vulgaris* in this experiment was comparable to those reported by other studies conducted on the genus Artemisia. The LC_50_ of *A. vulgaris* against *Ae. aegypti* larvae was lower than those of *A. nilagirica* and *A. annua* in 24 h and 48 h, respectively [9,19], benzene extract of *Ervatamia coronaria* and *Caesalpinia pulcherrima* [7], ethanolic extract of *Tribulus terrestris* [42], and nanoemulsion of *Vitex negundo* L essential oil [43]. The larvicidal LC_50_ of *A. vulgaris* extract against *Ae. aegypti* reported here is higher in 24 h exposure, i.e., 18.6 ppm than that of *A. vulgaris* in essential oil form, 4.269 ppm [44].

Insecticidal activity against adult *Ae. aegypti* has also been reported in other species of the genus *Artemisia*, for example, *A. nilagirica*, which demonstrated an LC_50_ 242.52 ppm [19]. *A. vulgaris* extract also showed deterrent, rather than attractant, activity against *Ae. aegypti* oviposition at the three tested concentrations (Table-1). At a concentration of 1000 ppm, *A. vulgaris* inhibited oviposition by 96%. The ovicidal assay demonstrated that *A. vulgaris* at a concentration of 1000 ppm resulted in 82.67% of eggs left unhatched; at 750 ppm, this figure was 79.33% and at 500 ppm, 44% (Figure-3). This result is similar to that reported for a 500 ppm concentration of *A. annua*, which resulted in a hatching rate of 48.84% [9]. The deterrent effect of *A. vulgaris* at 500 ppm was higher than that of *Aegle marmelos* (71.79%) and *Sphaeranthus amaranthoides* (8.74%), but lower than that of *Limonia acidissima* (100%) in the ethanolic extract [25].

The repellent activity of the extract tended to decrease over a longer exposure period, in contrast to that of the positive control. Many bioactive compounds with repellent activity are highly volatile [45]; therefore, non-controlled release formulations confer a shorter period of protection. The formation of complexes with nanoparticles, such as silver, has been evidenced to increase efficiency and stability over time [46].

This experiment clearly demonstrated the adulticidal, larvicidal, ovicidal, and deterrent properties of *A. vulgaris*. The efficacy of potential insecticidal compounds in oviposition and ovicidal assays should also be considered to avoid the possibility of transovarial dengue virus transmission in the vector. Bioactive plant compounds in whole-extract form may act synergistically to produce higher effectiveness than individual compounds acting alone [47]. The extraction process is low-cost, simple, and rapid. It has the potential to be effective as an additional substance in a commercial formulation, since *Ae. aegypti* is reportedly resistant to pyrethroid-based insecticide, which is commonly used by the Indonesian people [28]. The active components of *A. vulgaris*, i.e., camphor (26.99%), α-humulene (0.72%), β-caryophyllene (0.8%), and β-caryophyllene oxide (15.87%), have demonstrated strong insecticidal activity [44]. In addition, the administration of a plant-based formulation is relatively risk-free. Moreover, *A. vulgaris* is consumed orally by local citizens as a traditional herbal for fever, headache, and stomachache. To the best of our knowledge, there has been no toxicity reported from citizens consuming *A. vulgaris* so far [48].

## Conclusion

The development of resistance to chemical insecticides is growing rapidly worldwide. Natural herbs are considered as a promising new insecticide solution to control disease-carrying vectors. The effects of *A. vulgaris* in the stages of eggs and larvae can be developed further for bio-insecticides in water. The effectiveness of *A vulgaris* in the adult stage can be developed into a spray-type insecticide. *A. vulgaris* also may be used as cream or lotion for skin repellent. Taken together, these results indicate that *A. vulgaris* can be applied to repel or inhibit *Ae. aegypti* in various stages of its lifecycle. In addition, there have been no reports so far on the toxicity of *A. vulgaris* to mammals. Further research is necessary to design a delivery method to enhance its efficacy and stability as a bioinsecticide.

## Authors’ Contributions

ASM and NKF performed an experiment on the extraction procedures. VIN, EP, and ATU tested to mosquitoes and repellency assay. PHH and VIN analyzed the data and involved in the project administration. PHH, RW, and VIN coordinated the work and wrote the manuscript. All authors read and approved the final version of the manuscript.
